# Five new species of *Shaanxinus* Tanasevitch, 2006 (Araneae, Linyphiidae) from China

**DOI:** 10.3897/zookeys.1296.213068

**Published:** 2026-10-28

**Authors:** Muhammad Irfan, Tehreem Rehman, Lu-Yu Wang, Zhi-Sheng Zhang

**Affiliations:** 1 Key Laboratory of Eco-environments in Three Gorges Reservoir Region (Ministry of Education), School of Life Sciences, Southwest University, Chongqing 400715, China Key Laboratory of Eco-environments in Three Gorges Reservoir Region (Ministry of Education), School of Life Sciences, Southwest University Chongqing China; 2 College of Plant Protection, Southwest University, Chongqing 400715, China College of Plant Protection, Southwest University Chongqing China

**Keywords:** description, Erigoninae, morphology, taxonomy, sheet-web spider

## Abstract

Five new species of the genus *Shaanxinus* are described from Gansu, Guizhou, Shaanxi, and Sichuan Provinces, China: *S. baitianhe***sp. nov**., *S. kuankuoshui***sp. nov**., *S. liangdang***sp. nov**., *S. micangshan***sp. nov**., and *S. zhongzhai***sp. nov**. Detailed descriptions, photographs of the copulatory organs and somatic features of all five species, and a distribution map are provided. In addition, colour photographs of the body and genitalia of *Shaanxinus griseolineatus* (Schenkel, 1936) are presented. This study improves our understanding of the species diversity, distribution, and morphological variation of *Shaanxinus*, and underscores the importance of further integrative taxonomic research in this biologically diverse yet poorly sampled region.

## Introduction

The family Linyphiidae is one of the most diverse spider families worldwide, currently comprising 641 extant genera and 4,967 species, including 11 fossil genera and 62 fossil species (WSC 2026). In China, 639 species in 185 genera have been recorded (Tanasevitch 2026). Recently, about 51 species in 34 genera have been reported from Gansu, Guizhou, Shaanxi, and Sichuan Provinces (Irfan et al. 2025).

The genus *Shaanxinus* Tanasevitch, 2006 currently comprises 21 described species distributed in China (Chongqing, Hebei, Hunan, Shaanxi, and Taiwan), Russia (Far East), and Vietnam (WSC 2026). Lin et al. (2019) described 14 new species and provided the first comprehensive morphological comparisons with micro-CT examination of male prosomal glands and pedipalp structures, a morphological phylogenetic analysis, and COI, 16S, and 28S sequence data. It therefore provides an essential comparative framework for evaluating additional species, including variation in male cephalic structures and the genital characters used in species recognition. Despite recent taxonomic advances, the diversity and distribution of *Shaanxinus* remain insufficiently documented, particularly in mainland China and adjacent regions, indicating that additional undescribed species are likely to be discovered.

## Material and methods

The specimens were collected by sieving leaf litter and preserved in 75% ethanol. Left male pedipalps were examined and photographed. Epigynes were dissected, cleared in trypsin solution, and then examined and photographed. Specimens were examined and measured under a Leica M205A stereomicroscope equipped with a Leica DFC450 camera and LAS v. 4.6 software. Photographs of habitus and copulatory organs were taken with a Kuy Nice CCD camera mounted on an Olympus BX53 compound microscope. Focus-stacked images were generated using Helicon Focus v. 6.7.1. Eye sizes were measured as the maximum dorsal diameter. Leg measurements are presented as total length (femur, patella + tibia, metatarsus, tarsus). All measurements are given in millimetres. Specimens are deposited in the School of Life Sciences, Southwest University, Chongqing, China (**SWUC**). Genitalic terminology follows Lin et al. (2019).

The following abbreviations are used in the text: AER—anterior eye row; ALE—anterior lateral eyes; alt.—altitude; AME—anterior median eyes; AME–ALE—the distance between AME and ALE; AME–AME—the distance between AMEs; PER—posterior eye row; PLE—posterior lateral eyes; PME—posterior median eyes; PME–PLE—distance between PME and PLE; PME–PME—distance between PMEs.

## Taxonomy

### Family Linyphiidae Blackwall, 1859

**Subfamily Erigoninae Emerton, 1882**

#### Genus Shaanxinus

Taxon classificationAnimaliaAraneaeLinyphiidae

Tanasevitch, 2006

9A7CD2DF-E2C1-5E59-93F6-D437CE24CB15

##### Chinese name.

陕蛛属.

##### Type species.

*Shaanxinus rufus* Tanasevitch, 2006; gender masculine.

#### Shaanxinus baitianhe sp. nov.

Taxon classificationAnimaliaAraneaeLinyphiidae

DF790BAF-3F13-587D-9270-42955D379C9D

https://zoobank.org/C5AC4A4B-D6F1-43D8-AFBC-40ADC180DC1B

**Figure 1.**
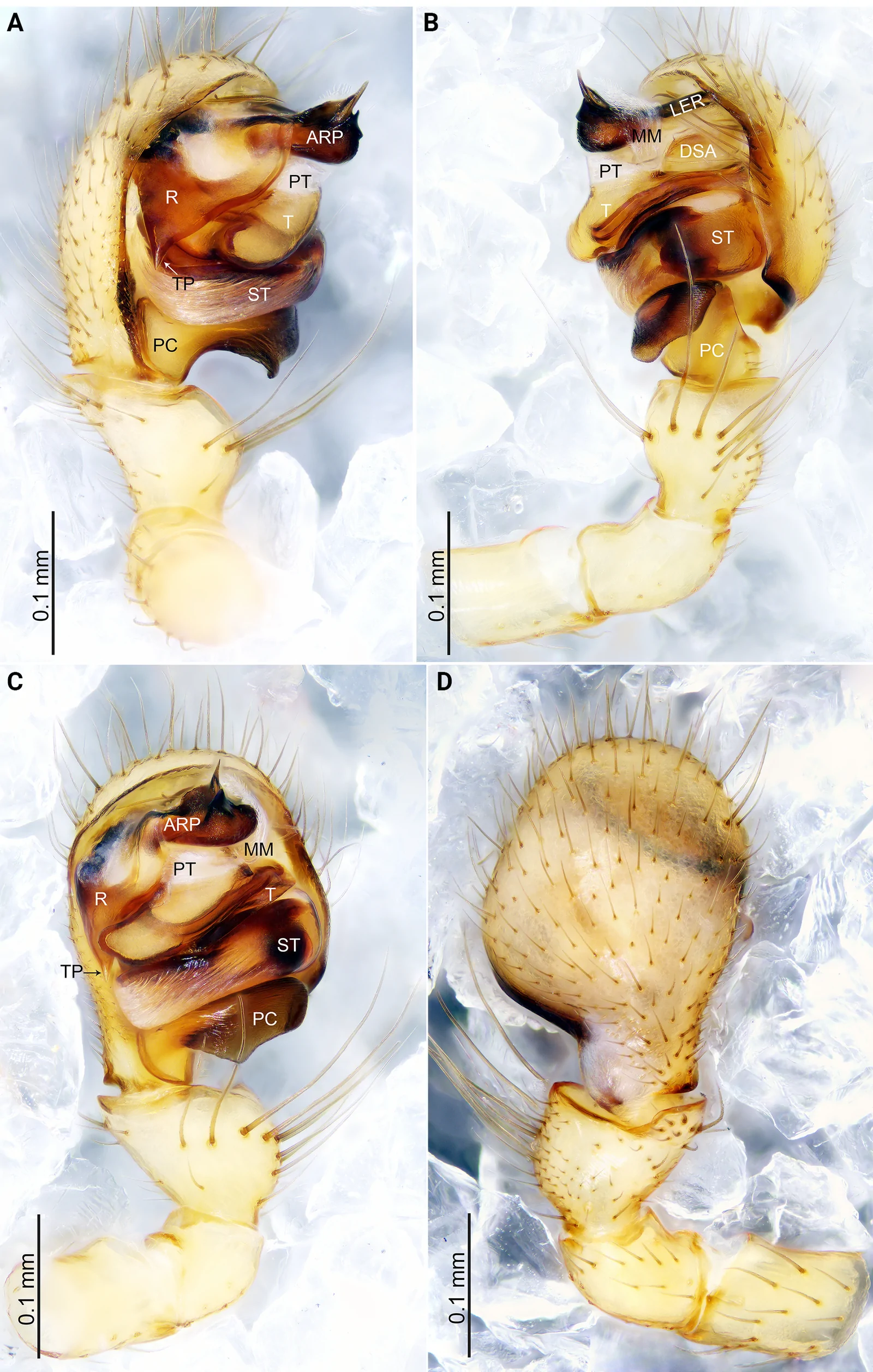
*Shaanxinus baitianhe* sp. nov., male holotype pedipalp. **A**. Prolateral view; **B**. Retrolateral view; **C**. Ventral view; **D**. Dorsal view. Abbreviations: ARP—Anterior radical process; DSA—distal suprategular apophysis; LER—lateral extension of radix; MM—median membrane; PC—paracymbium; PT—protegulum; R—radix; ST—subtegulum; T—tegulum; TP—tailpiece.

Figs 1A–D, 10A, 10B, 11

##### Chinese name.

白天河陕蛛.

##### Type material.

***Holotype***: ♂, China: • Shaanxi Province, Hanzhong City, Zhenba County, Qingshui Town, Renhe Village, Baitianhe Nature Reserve, 32°46.639'N, 107°42.942'E, alt. 1048 m, 2013-5-26, Liu Mingxin, Meng Xiangwei leg. (SWUC-T-LIN-40-01).

##### Etymology.

The specific epithet is derived from the type locality.

##### Diagnosis.

Male of *Shaanxinus baitianhe* sp. nov. resembles *S. rufus* Tanasevitch, 2006 in having a similar paracymbium (Fig. 1B; Lin et al. 2019: fig. 7b), but it can be distinguished by the anterior radical process apex with one long projection and two small teeth (Fig. 1A), which are absent in *S. rufus* (Tanasevitch 2006: fig. 51).

##### Description.

**Male** (holotype, Fig. 10A, B): total length: 3.45. Carapace 1.63 long, 1.19 wide, yellow, cephalic lobe 0.81 high; sulci present below anterior eye row; fovea, cervical and radial grooves distinct. Clypeus 0.37 high, with many microsetae. Chelicerae with 5 promarginal and 4 retromarginal teeth. AER strongly recurved, PER procurved, slightly wider than AER. Eye sizes and interdistances: AME 0.05, ALE 0.06, PME 0.06, PLE 0.07, AME–AME 0.06, PME–PME 0.12, AME–PME 0.15, AME–ALE 0.12, PME–PLE 0.12, ALE–ALE 0.46, PLE–PLE 0.54, ALE–PLE 0.02. Length of legs: I 5.25 (1.63, 1.82, 1.20, 0.60), II 5.86 (1.95, 1.99, 1.31, 0.61), III 4.09 (1.25, 1.31, 1.06, 0.47), IV lost. Leg II longer than I. TmI 0.32. Tibial spine formula: 2–2–2–0. Abdomen 1.87 long, 1.18 wide, oval, pale, greenish-black pattern, ventral side pale.

Pedipalp (holotype, Fig. 1A–D). Patella as long as tibia, ventrally grooved. Tibia with 2 retrolateral and 1 dorsal trichobothria, posterior margin with long, thick spines. Paracymbium C-shaped, distal arm tip margin wave-like in retrolateral view. Tegulum extending retrolaterally, with well-developed membranous protegulum. Distal suprategular apophysis disc-shaped, tapering towards tip. Radix with large anterior radical scaly part with 2 small teeth; anterior radical process distal end sharp projection with pointed tip in prolateral view; lateral extension of radix slightly curved, apex blunt in retrolateral view, covering terminal part of embolus; tailpiece triangular with pointed end; median membrane present; embolus sclerotised, arc-shaped.

**Female**. Unknown.

##### Distribution.

Known only from the type locality (Fig. 11).

#### Shaanxinus griseolineatus

Taxon classificationAnimaliaAraneaeLinyphiidae

(Schenkel, 1936)

4A7197A4-48A6-5E3C-8DF3-8CA70B36EC4D

**Figure 2.**
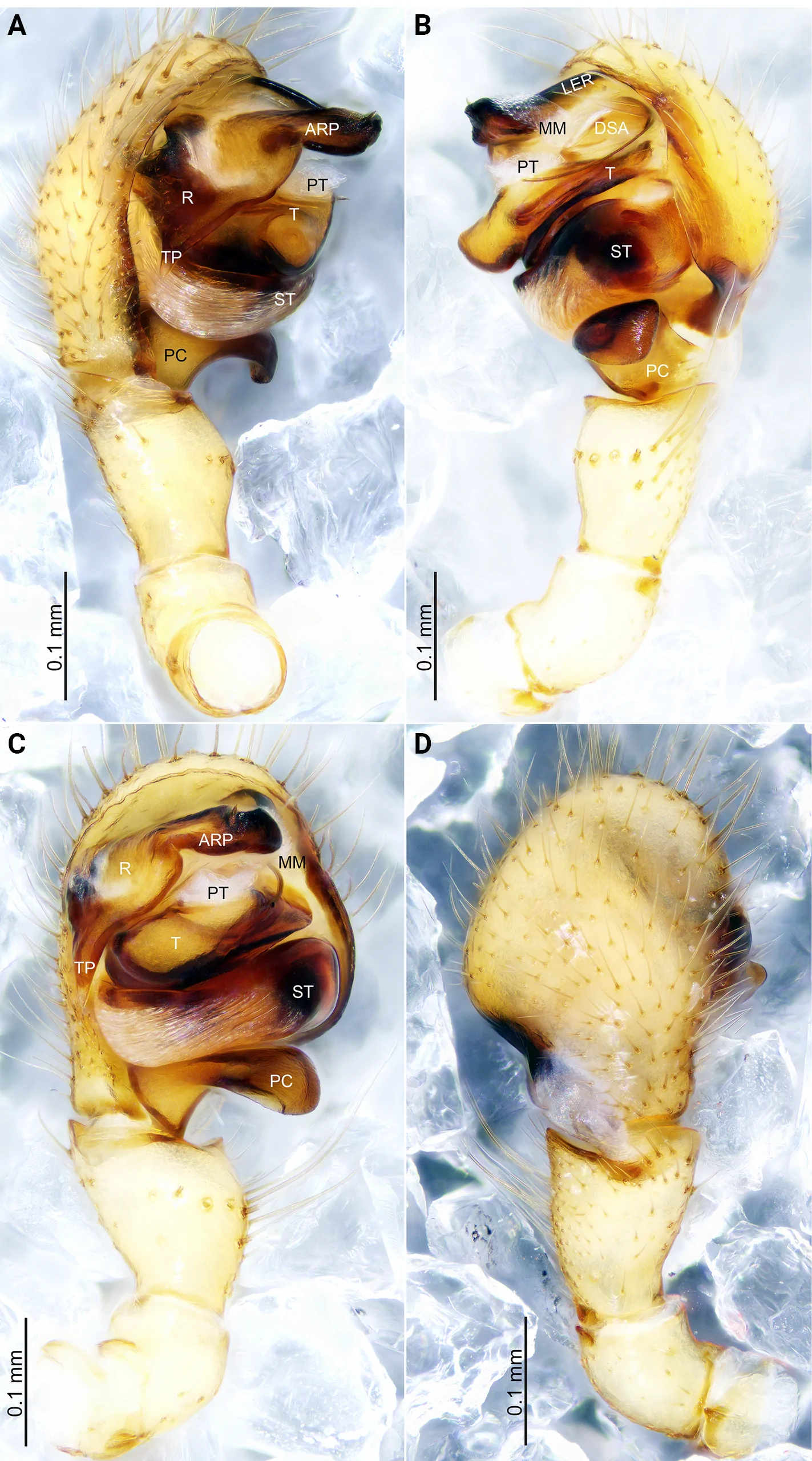
*Shaanxinus griseolineatus* (Schenkel, 1936), male pedipalp. **A**. Prolateral view; **B**. Retrolateral view; **C**. Ventral view; **D**. Dorsal view. Abbreviations: ARP—Anterior radical process; DSA—distal suprategular apophysis; LER—lateral extension of radix; MM—median membrane; PC—paracymbium; PT—protegulum; R—radix; ST—subtegulum; T—tegulum; TP—tailpiece.

**Figure 3.**
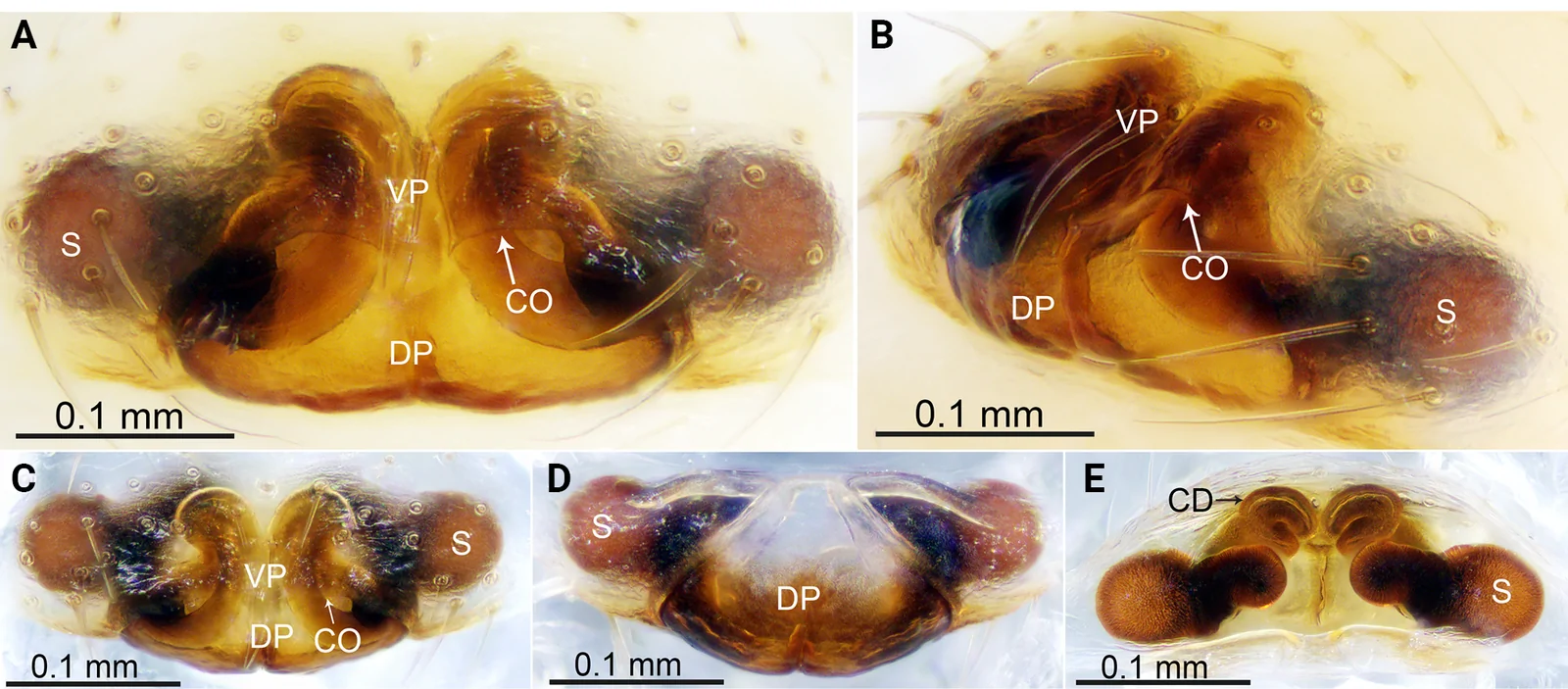
*Shaanxinus griseolineatus* (Schenkel, 1936), female epigyne. **A, C**. Ventral view; **B**. Lateral view; **D**. Dorsal view; **E**. Anterior view. Abbreviations: CD—copulatory duct; CO—copulatory opening; DP—dorsal plate; FD—fertilisation duct; S—spermathecae; VP—ventral plate.

Figs 2A–D, 3A–E, 10C–E, 11

Gonatium griseolineatum Schenkel, 1936: 58, fig. 13 (described female).
Oinia griseolineata —Tanasevitch 1989: 170, fig. 222 (female transferred from Gonatium).
Gongylidioides griseolineatus —Eskov 1992: 159 (transferred from Oinia); Song et al. 1999: 170, fig. 97I (female); Tu and Li 2006: 59, fig. 4A–F (female); Zuo et al. 2020: 27, fig. 1A–G (described male and female).
Shaanxinus griseolineatus —Irfan et al. 2025: 167 (transferred from Gongylidioides).

##### Chinese name.

灰线陕蛛.

##### Material examined.

• 5♂3♀, **China, Gansu Province**, Liangdang County, Yangdian Township, Yangdian Village, 33°56.320'N, 106°24.480'E, alt. 1004 m, 2011-5-31, Wang Luyu leg.; • 4♂1♀, **Shaanxi Province**, Zhouzhi County, Houzhenzi Old County Town, 33°47.357'N, 107°45.734'E, alt. 1667 m, 2010-10-2, Li Zongxu, Li Zhong, Wang Luyu leg.; • 1♀, **Sichuan Province**, Guangyuan City, Qingchuan County, Qingxi Town, Baiguoping Baohu Station, 32°32.649'N, 104°49.680'E, alt. 1171 m, Meng Xiangwei, Zhao Li, Yang Juncai leg.; • 1♂1♀, Wanyuan City, Hua’e Township, Hua’e Mountain, 32°04.082'N, 108°10.629'E, alt. 1144 m, 2013-5-23, Liu Mingxin, Meng Xiangwei leg.

##### Distribution.

China (Gansu, Hebei, Henan, Shaanxi, Shandong and Sichuan, Fig. 11) and Russia (Far East).

#### Shaanxinus kuankuoshui sp. nov.

Taxon classificationAnimaliaAraneaeLinyphiidae

72CCAAE5-105E-5208-9D3E-291CFE43B833

https://zoobank.org/660805EA-87B3-4174-A5DA-6541CD94F67D

**Figure 4.**
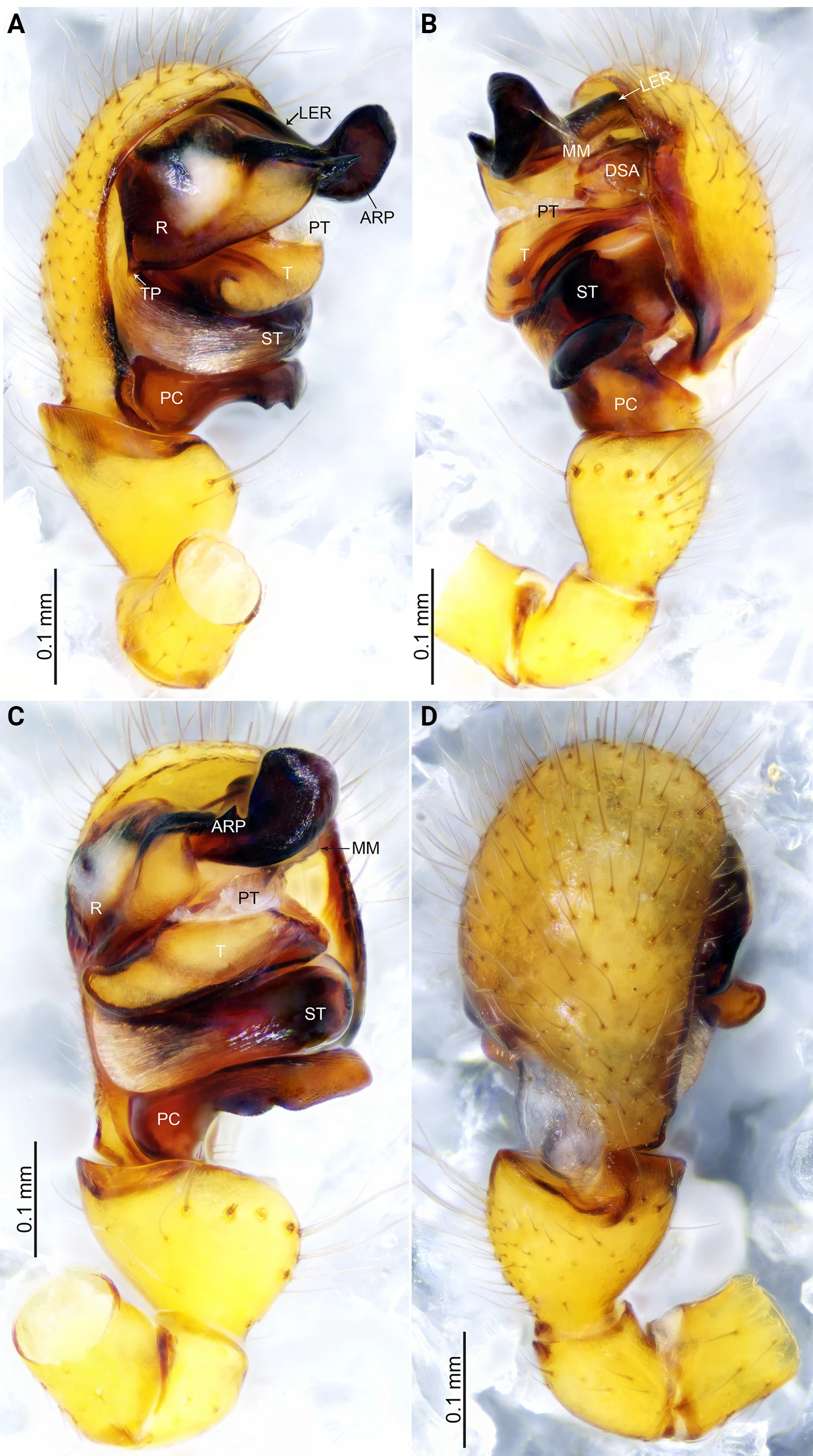
*Shaanxinus kuankuoshui* sp. nov., male holotype pedipalp. **A**. Prolateral view; **B**. Retrolateral view; **C**. Ventral view; **D**. Dorsal view. Abbreviations: ARP—Anterior radical process; DSA—distal suprategular apophysis; LER—lateral extension of radix; MM—median membrane; PC—paracymbium; PT—protegulum; R—radix; ST—subtegulum; T—tegulum; TP—tailpiece.

Figs 4A–D, 10F, 10G, 11

##### Chinese name.

宽阔水陕蛛.

##### Type material.

***Holotype***: • ♂, China: Guizhou Province, Kuankuoshui Reservoir, Kuankuoshui Nature Reserve Central Management Station, 28°12.473'N, 107°10.378'E, alt. 1435 m, 2010-06-04, Li Zongxu, Wang Luyu leg. (SWUC-T-LIN-41-01).

##### Etymology.

The specific epithet is derived from the type locality.

##### Diagnosis.

Male of *Shaanxinus kuankuoshui* sp. nov. resembles *S. longiembolus* Irfan, Zhang & Peng, 2025 in having a similar paracymbium and anterior radical apophysis (Fig. 4B; Irfan et al. 2025: fig. 218), but it can be distinguished by the lateral extension of radix tip being arc-shaped in retrolateral view (Fig. 4B), vs thumb-shaped in *S. longiembolus* (Irfan et al. 2025: fig. 218B).

##### Description.

**Male** (holotype, Fig. 10F, G): total length: 3.09. Carapace 1.49 long, 1.08 wide, yellowish brown, cephalic lobe 0.60 high with many microsetae; sulci present below the anterior eye row; fovea, cervical and radial grooves distinct. Clypeus 0.37 high. Chelicerae with 5 promarginal and 4 retromarginal teeth. AER recurved, PER straight, slightly wider than AER. Eye sizes and interdistances: AME 0.05, ALE 0.08, PME 0.08, PLE 0.08, AME–AME 0.03, PME–PME 0.10, AME–PME 0.07, AME–ALE 0.07, PME–PLE 0.06, ALE–ALE 0.37, PLE–PLE 0.41, ALE–PLE 0.01. Length of legs: I 4.99 (1.49, 1.72, 1.15, 0.63), II 5.15 (1.52, 1.68, 1.31, 0.64), III 3.37 (1.11, 1.10, 0.78, 0.38), IV 4.33 (1.25, 1.31, 1.16, 0.61). Leg II longer than I. TmI 0.26. Tibial spine formula: 2-2-2-2. Abdomen 1.49 long, 1.10 wide, oval, grey dorsally with 6 transverse chevrons, ventral side black.

Pedipalp (holotype, Fig. 4A–D). Patella slightly shorter than tibia, ventrally grooved. Tibia with 2 retrolateral and 1 dorsal trichobothria, posterior margin with long, thick spines. Paracymbium C-shaped, distal arm margin wavy in retrolateral view. Tegulum extending retrolaterally, possessing a small membranous protegulum. Distal suprategular apophysis disc-shaped, with bifurcated end. Radix with anterior radical process small, strongly sclerotised with pointed end; lateral extension of radix slightly curved, apical end robust arc-shaped projection in retrolateral view; tailpiece small, triangular, with pointed end; median membrane present; embolus sclerotised, semicircular, terminal part ends in the apex of lateral extension of radix.

**Female**. Unknown.

##### Distribution.

Known only from the type locality (Fig. 11).

#### Shaanxinus liangdang sp. nov.

Taxon classificationAnimaliaAraneaeLinyphiidae

444E2AA4-1AE3-5D51-A732-ACB4E10FD2E0

https://zoobank.org/CC903C8C-04B5-4AAF-ADF6-6791F6889246

**Figure 5.**
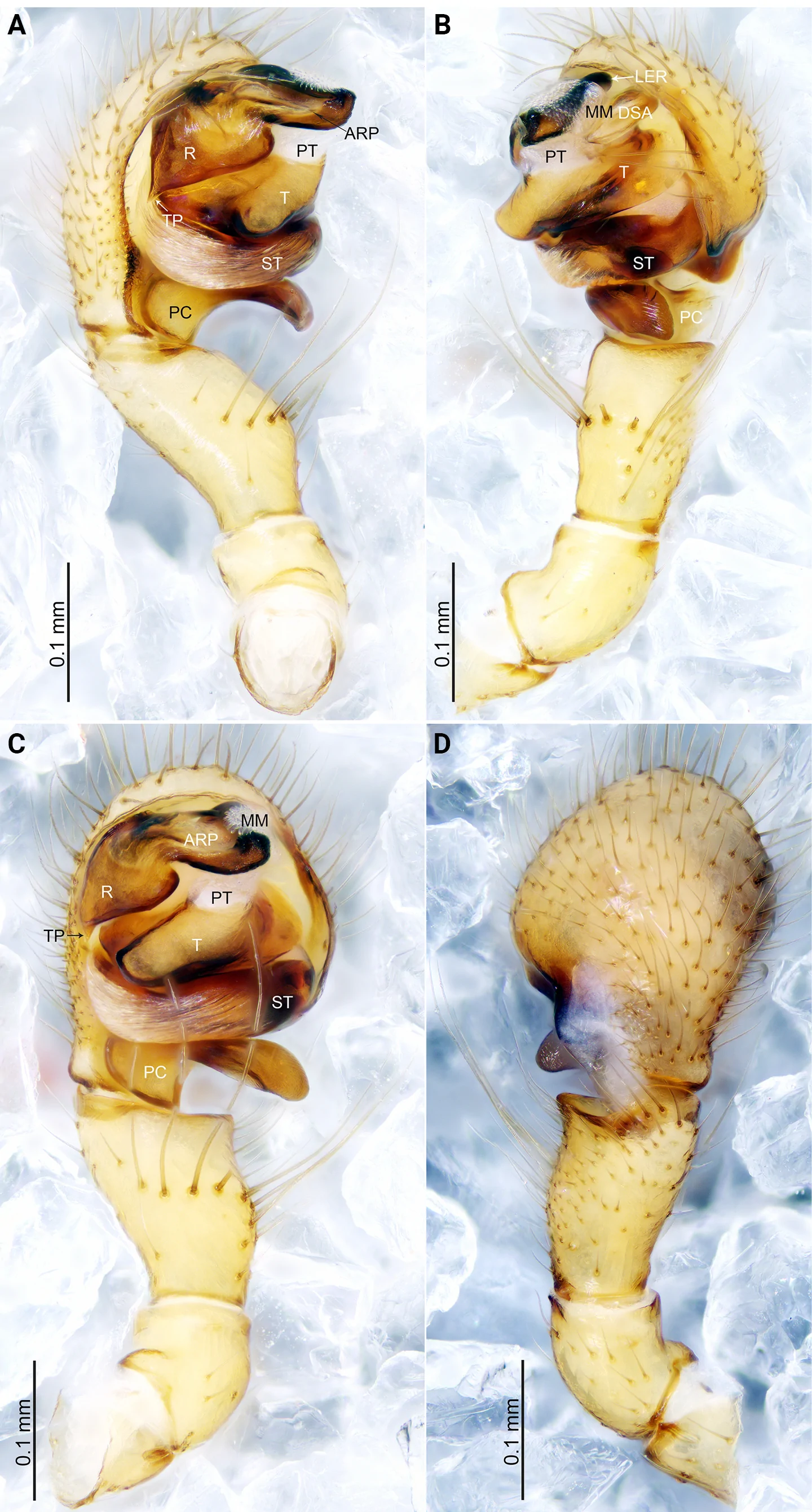
*Shaanxinus liangdang* sp. nov., male holotype pedipalp. **A**. Prolateral view; **B**. Retrolateral view; **C**. Ventral view; **D**. Dorsal view. Abbreviations: ARP—Anterior radical process; DSA—distal suprategular apophysis; LER—lateral extension of radix; MM—median membrane; PC—paracymbium; PT—protegulum; R—radix; ST—subtegulum; T—tegulum; TP—tailpiece.

**Figure 6.**
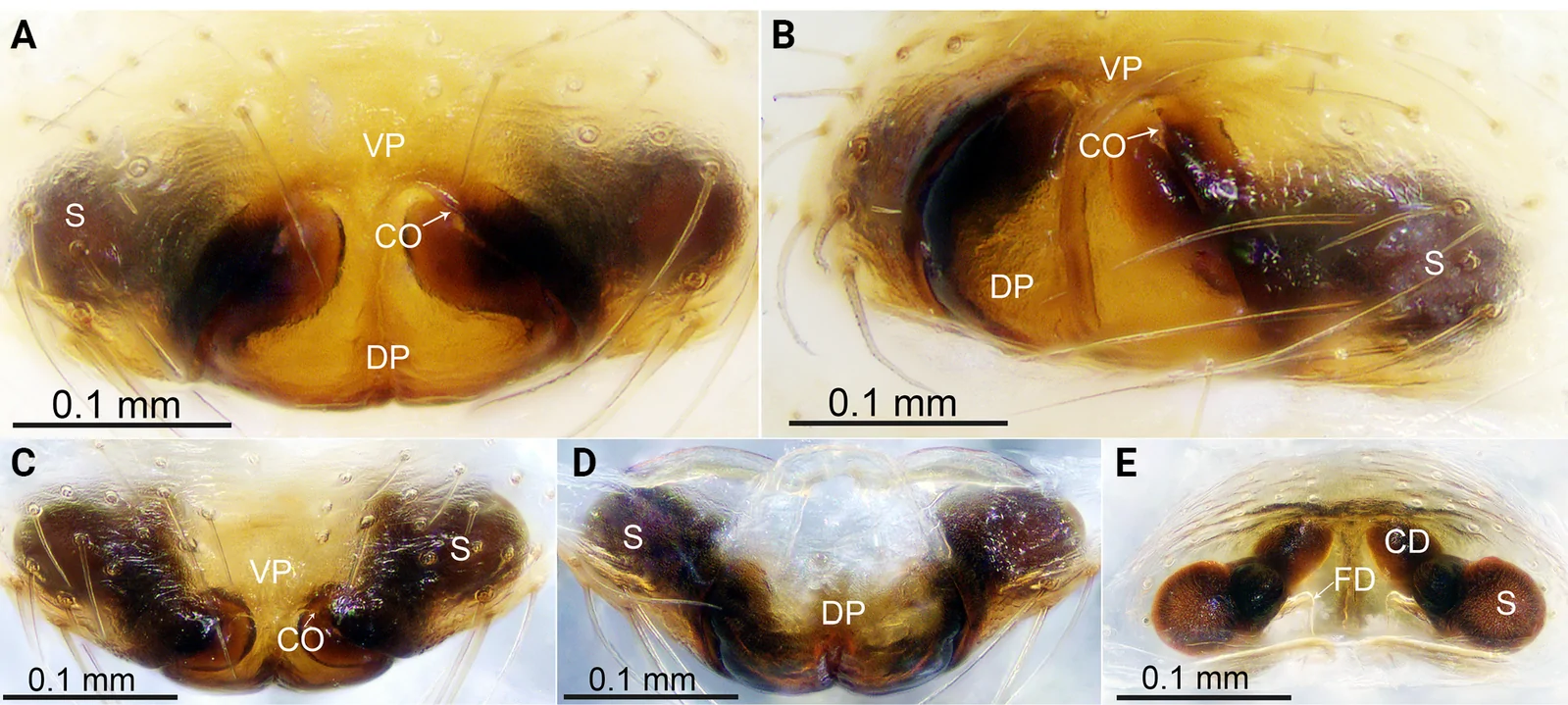
*Shaanxinus liangdang* sp. nov., female paratype epigyne. **A, C**. Ventral view; **B**. Lateral view; **D**. Dorsal view; **E**. Anterior view. Abbreviations: CD—copulatory duct; CO—copulatory opening; DP—dorsal plate; FD—fertilisation duct; S—spermathecae; VP—ventral plate.

Figs 5A–D, 6A–E, 10H–J, 11

##### Chinese name.

两当陕蛛.

##### Type material.

***Holotype***: • 1♂, China, **Gansu Province**: Liangdang County, Zhangjia Township, Heihe Forest Park, 34°11.020'N, 106°32.300'E, alt. 1774 m, 2011-06-1, Wang Luyu leg. (SWUC-T-LIN-42-01). ***Paratypes*** • (28♂27♀): 2♂2♀, same data as holotype male (SWUC-T-LIN-42-02–05); • 1♂, Liangdang County, Yangdian Township, Yangdian Village, 33°56.320'N, 106°24.480'E, alt. 1004 m, 2011-05-31, Wang Luyu leg. (SWUC-T-LIN-42-06); • 3♂1♀, **Shaanxi Province**: Taibai Mountain, Benyue Old County Town, 33°47.544'N, 107°45.594'E, alt. 1761 m, 2009.05.25, Wang Luyu leg. (SWUC-T-LIN-42-07–10); • 4♂5♀, southern slope of Taibai Mountain (Zhou Zhihou’s site), 33°52.477'N, 107°48.814'E, alt. 1577 m, 2009.05.24, Wang Luyu leg. (SWUC-T-LIN-42-11–19); • 1♂, 33°52.954'N, 107°48.722'E, alt. 1577 m, 2009.05.24, Wang Luyu leg. (SWUC-T-LIN-42-20); • 9♂12♀, southern slope of Taibai Mountain (Zhou Zhihou’s site), 33°53.820'N, 107°47.946'E, alt. 1979 m, 2009.05.24, Wang Luyu leg. (SWUC-T-LIN-42-21–41); • 8♂7♀, Zhouzhi County, Juhouzhenzi, Back slope of Qingfenglou Farmhouse, 33°53.665'N, 107°48.198'E, alt. 1935 m, Li Zonggui, Xu Kaiyi, Wang Luyu leg. (SWUC-T-LIN-42-42–56).

##### Etymology.

The specific epithet is derived from the type locality.

##### Diagnosis.

**Figure 7.**
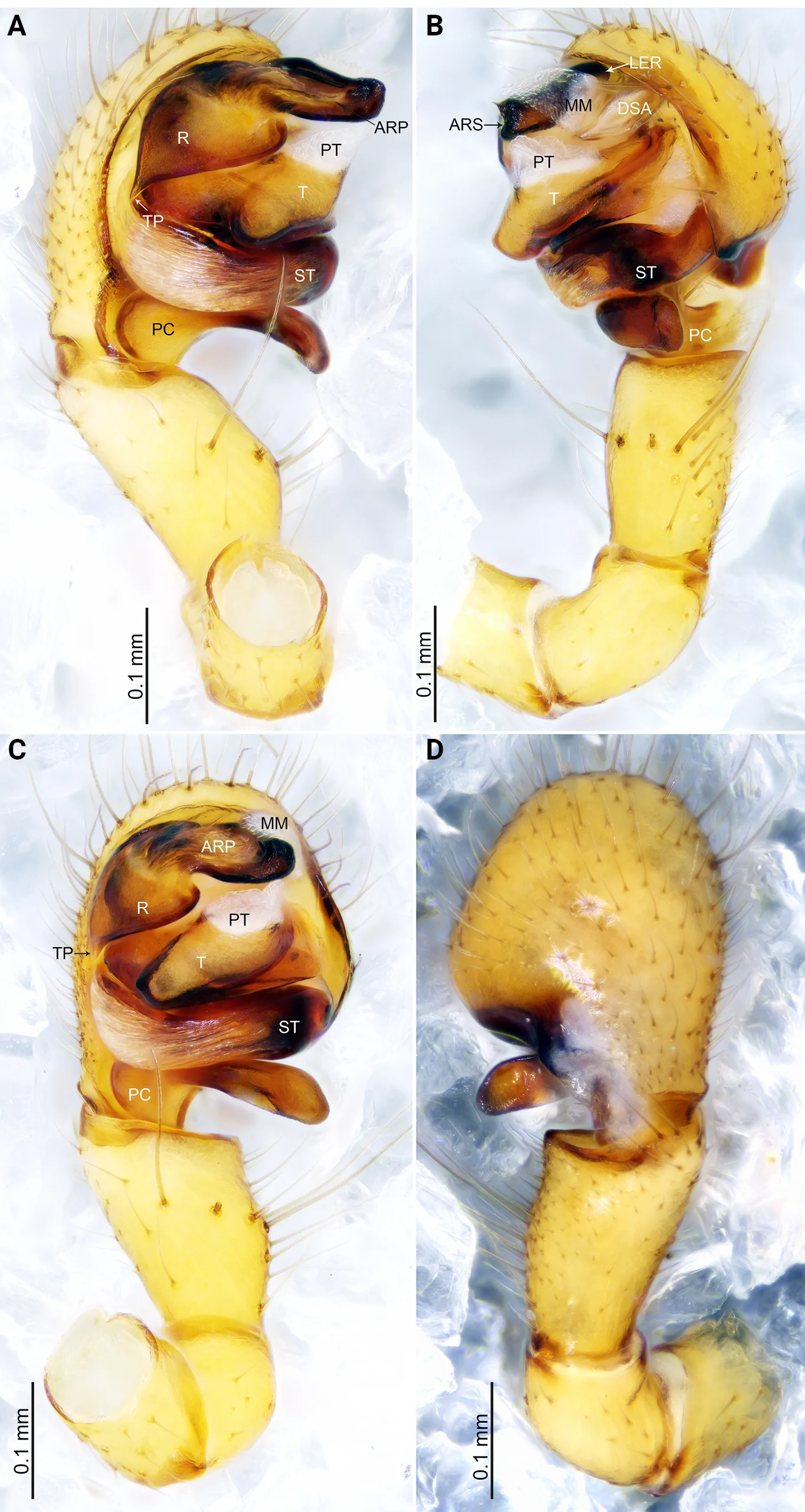
*Shaanxinus micangshan* sp. nov., male holotype pedipalp. **A**. Prolateral view; **B**. Retrolateral view; **C**. Ventral view; **D**. Dorsal view. Abbreviations: ARP—Anterior radical process; ARS—Anterior radical scaly part; DSA—distal suprategular apophysis; LER—lateral extension of radix; MM—median membrane; PC—paracymbium; PT—protegulum; R—radix; ST—subtegulum; T—tegulum; TP—tailpiece.

The male of *Shaanxinus liangdang* sp. nov. resembles *S. micangshan* sp. nov. in having a similar embolic division (Figs 5A, 7A), but it can be distinguished by the anterior radical apophysis tip lacking a projection (Fig. 5B), vs with a triangular projection in *S. micangshan* sp. nov. (Fig. 7B); the distal arm of paracymbium covering more than half of the proximal part, with the tip tapering in retrolateral view (Fig. 5B), vs the distal arm covering half of the proximal part and the tip margin straight in *S. micangshan* sp. nov. (Fig. 7B). The epigyne of *S. liangdang* sp. nov. resembles *S. shoukaensis* Lin, 2019 in having a reduced ventral plate (Fig. 6A–E; Lin et al. 2019: fig. 15f–h), but it is distinguished by having the spermathecae pointing laterally at the centre (Fig. 6A), not pointing towards the epigastric furrow in *S. shoukaensis* (Lin et al. 2019: fig. 15f), and the copulatory ducts form a broad loop in ventral view (Fig. 6A), rather than a narrow loop in *S. shoukaensis* (Lin et al. 2019: fig. 15f).

##### Description.

**Male** (holotype, Fig. 10H, I): total length: 3.12. Carapace 1.44 long, 1.17 wide, yellow, cephalic lobe 0.65 high with many microsetae; sulci present below the anterior eye row; fovea, cervical and radial grooves distinct. Clypeus 0.46 high. Chelicerae with 5 promarginal and 4 retromarginal teeth. AER strongly recurved, PER straight, slightly wider than AER. Eye sizes and interdistances: AME 0.07, ALE 0.06, PME 0.07, PLE 0.07, AME–AME 0.06, PME–PME 0.08, AME–PME 0.08, AME–ALE 0.03, PME–PLE 0.03, ALE–ALE 0.27, PLE–PLE 0.32, ALE–PLE 0.02. Length of legs: I 4.84 (1.43, 1.67, 1.05, 0.69), II 5.35 (1.51, 1.86, 1.40, 0.58), III 4.44 (1.24, 1.38, 1.30, 0.52), IV 4.95 (1.37, 1.63, 1.33, 0.62). Leg II longer than I. TmI 0.34. Tibial spine formula: 2-2-2-2. Abdomen 1.73 long, 1.13 wide, oval, pale, greenish-grey pattern followed by five transverse chevrons, ventral side greyish-pale.

Pedipalp (holotype, Fig. 5A–D). Patella 2/3 length of tibia, ventrally grooved. Tibia long, with 2 retrolateral and 1 dorsal trichobothria, with long, thick spines medially. Paracymbium U-shaped, distal arm covers more than half of proximal part in retrolateral view, tip tapering. Tegulum extending retrolaterally, with membranous protegulum. Distal suprategular apophysis disc-shaped, apically tapering. Radix with large, conspicuous anterior radical scaly part; anterior radical process membranous; lateral extension of radix slightly curved, ventrally grooved, wrapping embolus; tailpiece triangular, with pointed end; median membrane present; embolus sclerotised, arc-shaped, terminal part ends in tip of lateral extension of radix.

**Female** (paratype, Fig. 10J): total length: 3.71. Carapace 1.35 long, 1.05 wide. Clypeus 0.28 high. Chelicerae with 6 promarginal and 5 retromarginal teeth. AER recurved, PER straight, slightly wider than AER. Eye sizes and interdistances: AME 0.07, ALE 0.07, PME 0.07, PLE 0.08, AME–AME 0.03, PME–PME 0.07, AME–PME 0.07, AME–ALE 0.02, PME–PLE 0.03, ALE–ALE 0.29, PLE–PLE 0.35, ALE–PLE 0.02. Length of legs: I 5.14 (1.39, 1.73, 1.33, 0.69), II 5.26 (1.43, 1.73, 1.39, 0.71), III 3.92 (1.17, 1.19, 1.03, 0.53), IV 4.88 (1.38, 1.48, 1.33, 0.69). TmI 0.23. Abdomen 2.54 long, 2.04 wide. All other morphological characters same as in the male.

Epigyne (Fig. 6A–E). Epigynal plate wider than long. Ventral plate not extended posteriorly. Dorsal plate visible in ventral view, posterior margin with median depression. Copulatory openings present anteriorly. Vulva: copulatory ducts forming 2 broad loops before entering spermathecae. Spermathecae globular, dorso-laterally located, separated by more than twice their diameter; fertilisation ducts large, mesally extending.

##### Distribution.

Known only from the type locality (Fig. 11).

#### Shaanxinus micangshan sp. nov.

Taxon classificationAnimaliaAraneaeLinyphiidae

F54FB828-2F47-508B-814D-294D224ED0FD

https://zoobank.org/B46AB1E3-E783-463E-BC6F-3DECA561BCE3

**Figure 8.**
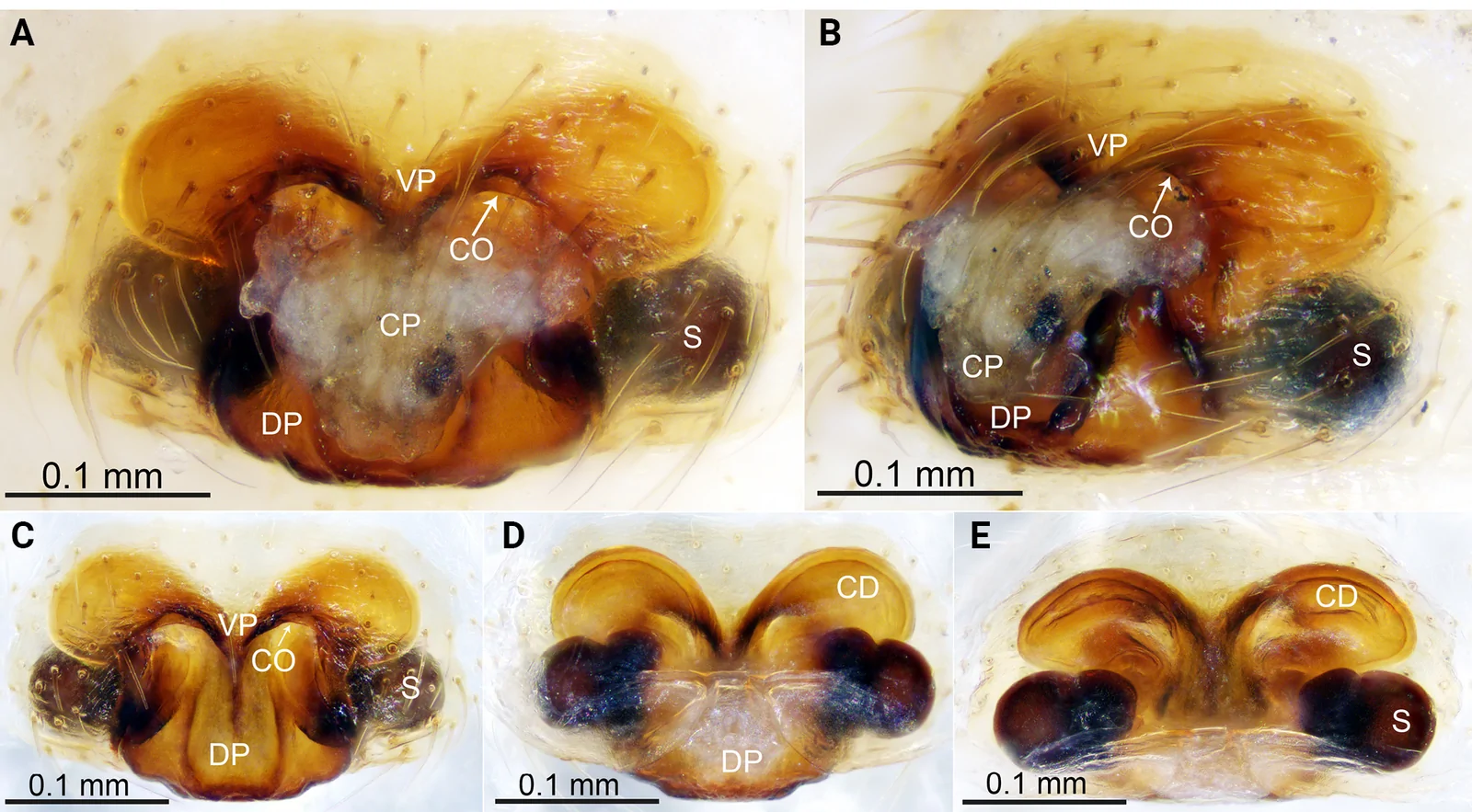
*Shaanxinus micangshan* sp. nov., female paratype epigyne. **A, C**. Ventral view; **B**. Lateral view; **D**. Dorsal view; **E**. Anterior view. Abbreviations: CD—copulatory duct; CO—copulatory opening; CP—copulatory plug; DP—dorsal plate; FD—fertilisation duct; S—spermathecae; VP—ventral plate.

Figs 7A–D, 8A–E, 10K–M, 11

##### Chinese name.

米仓山陕蛛.

##### Type material.

***Holotype***: • ♂, China: Sichuan Province, Nanjiang County, Micang Mountain, Daxiaolangou, 32°39.981'N, 106°54.759'E, alt. 1368 m, Jiang Xuankong, Wang Dong leg. (SWUC-T-LIN-43-01). ***Paratypes*** • (3♂3♀): 1♀, same data as holotype male (SWUC-T-LIN-43-02); • 3♂1♀, Aba Tibetan and Qiang Autonomous Prefecture, Li County, Shangmeng Township, Mengdonggou, 31°42'35.20"N, 103°7'51.16"E, alt. 2511 m, 2026-06-15, Muhammad Irfan, Cai Yujun, Tehreem Rehman, Shang Junxuan, and Zhou Hanping leg. (SWUC-T-LIN-43-03–06); • 1♀, Kangding City, Kongyu Township, Selong Village, 30°33'0.0600"N, 102°5'2.5740"E, alt. 2480.3 m, 2026-08-05, Muhammad Irfan, Cheng Linxin, Tehreem Rehman and Zhou Hanping leg. (SWUC-T-LIN-43-07).

##### Etymology.

The specific epithet is derived from the type locality.

##### Diagnosis.

Male of *Shaanxinus micangshan* sp. nov. resembles *S. liangdang* sp. nov. in having a similar embolic division (Figs 7A, 5A), but it can be distinguished by having the anterior radical apophysis tip with a triangular projection (Fig. 7B), vs without a projection in *S. liangdang* sp. nov. (Fig. 5B), the distal arm of the paracymbium covering half of the proximal part and the tip margin straight in retrolateral view (Fig. 7B), vs the distal arm covering more than half of the proximal part and the tip tapering in *S. liangdang* sp. nov. (Fig. 5B). The epigyne of *S. micangshan* sp. nov. can be distinguished from that of all other congeners by the balloon-shaped proximal part of the copulatory ducts and the ventrally grooved dorsal plate (Fig. 8A, C).

##### Description.

**Male** (holotype, Fig. 10K, L): total length: 3.31. Carapace 1.48 long, 1.23 wide, yellowish brown, cephalic lobe 0.51 high with many microsetae; sulci present below the anterior eye row; fovea, cervical and radial grooves distinct. Clypeus 0.45 high. Chelicerae with 5 promarginal and 4 retromarginal teeth. AER strongly recurved, PER straight, slightly wider than AER. Eye sizes and interdistances: AME 0.06, ALE 0.05, PME 0.06, PLE 0.06, AME–AME 0.07, PME–PME 0.08, AME–PME 0.11, AME–ALE 0.04, PME–PLE 0.05, ALE–ALE 0.26, PLE–PLE 0.31, ALE–PLE 0.03. Length of legs: I 5.44 (1.59, 1.85, 1.32, 0.68), II 5.87 (1.63, 1.96, 1.56,0.72), III 4.65 (1.35, 1.42, 1.31, 0.57), IV 6.07 (1.79, 1.99, 1.58, 0.71). Leg IV longer than II. TmI 0.37. Tibial spine formula: 2-2-2-2. Abdomen 1.94 long, 0.99 wide, oval, grey, blackish pattern followed by five transverse chevrons, ventral side grey.

Pedipalp (holotype, Fig. 7A–D). Patella 2/3 length of tibia, ventrally grooved. Tibia long, with 2 retrolateral and 1 dorsal trichobothria, medially with long, thick spines. Paracymbium U-shaped, distal arm covers half of proximal part in retrolateral view, tip margin straight. Tegulum extending retrolaterally, with membranous protegulum. Distal suprategular apophysis disc-shaped, with tapering tip. Radix with membranous anterior radical process; lateral extension of radix slightly curved, ventrally grooved, wrapping embolus, triangular projection with apical pointed end; tailpiece triangular, with pointed end; median membrane present; embolus sclerotised, arc-shaped, terminal part ends in tip of lateral extension of radix.

**Female** (paratype, Fig. 10M): total length: 3.37. Carapace 1.47 long, 1.04 wide. Clypeus 0.31 high. Chelicerae with 6 promarginal and 5 retromarginal teeth. AER recurved, PER procurved, slightly wider than AER. Eye sizes and interdistances: AME 0.05, ALE 0.07, PME 0.07, PLE 0.07, AME–AME 0.05, PME–PME 0.09, AME–PME 0.07, AME–ALE 0.04, PME–PLE 0.04, ALE–ALE 0.34, PLE–PLE 0.41, ALE–PLE 0.01. Length of legs: I 5.05 (1.51, 1.72, 1.24, 0.58), II 5.23 (1.56, 1.84, 1.25, 0.58), III 3.62 (1.11, 1.11, 0.99, 0.41), IV 4.73 (1.31, 1.51, 1.35, 0.56). TmI 0.23. Abdomen 2.15 long, 1.41 wide. All other morphological characters same as in the male, except for the pale colour.

Epigyne (Fig. 8A–E). Epigynal plate wider than long. Ventral plate not extended posteriorly. Dorsal plate visible, ventrally grooved, posterior margin wavy. Copulatory openings situated anteriorly beneath ventral plate. Vulva: copulatory ducts anteriorly forming broad, balloon-like loop, then turning dorsally. Spermathecae globular, dorso-lateral, separated by twice their diameter; fertilisation ducts large, mesally extending.

##### Distribution.

Known only from the type locality (Fig. 11).

#### Shaanxinus zhongzhai sp. nov.

Taxon classificationAnimaliaAraneaeLinyphiidae

DB1BB44D-B21B-56BF-AD0E-3B5CE6B04428

https://zoobank.org/40E7661F-4828-480A-9B86-B7B68C7F53BD

**Figure 9.**
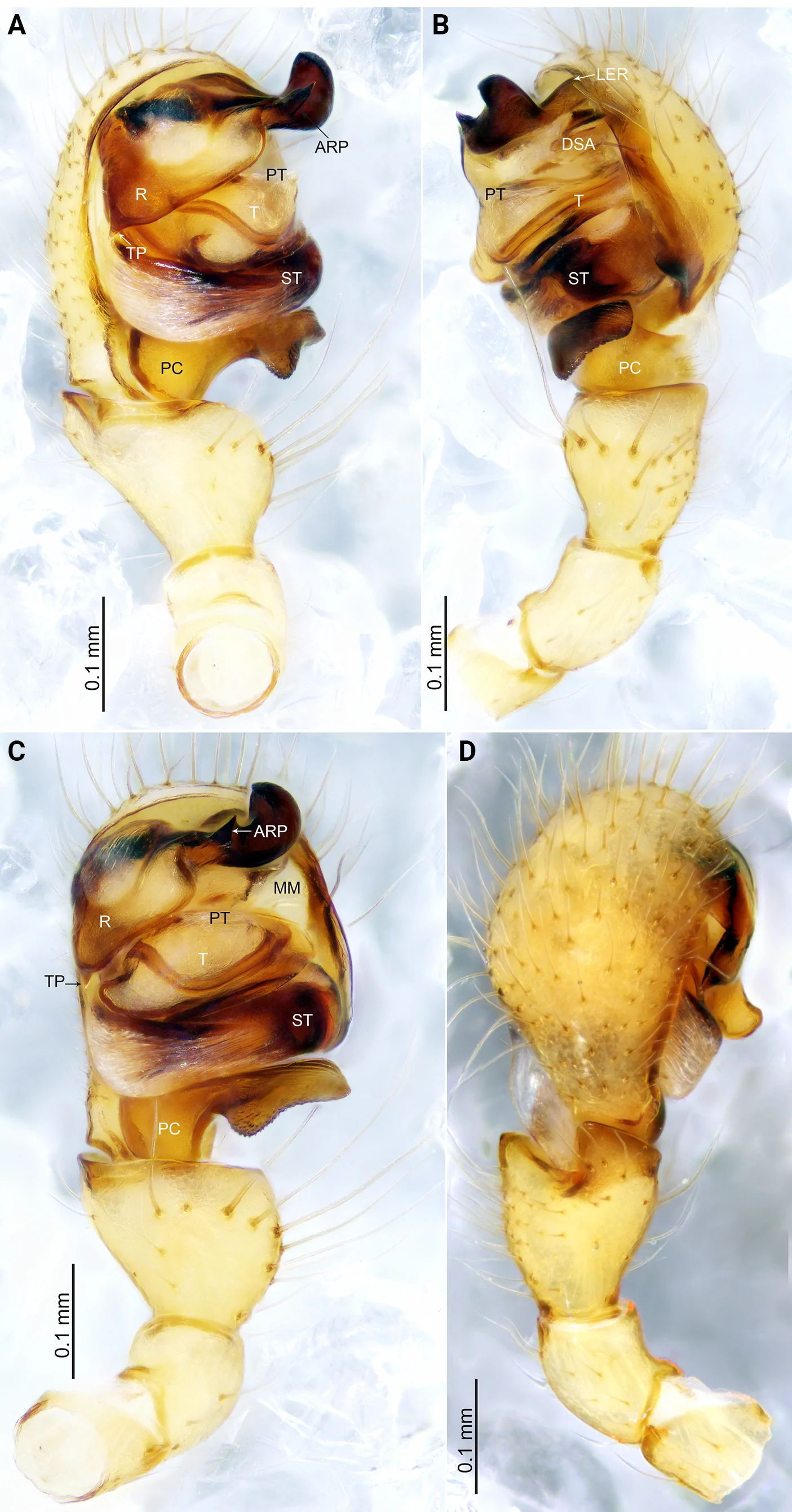
*Shaanxinus zhongzhai* sp. nov., male holotype pedipalp. **A**. Prolateral view; **B**. Retrolateral view; **C**. Ventral view; **D**. Dorsal view. Abbreviations: ARP—Anterior radical process; DSA—distal suprategular apophysis; LER—lateral extension of radix; MM—median membrane; PC—paracymbium; PT—protegulum; R—radix; ST—subtegulum; T—tegulum; TP—tailpiece.

**Figure 10.**
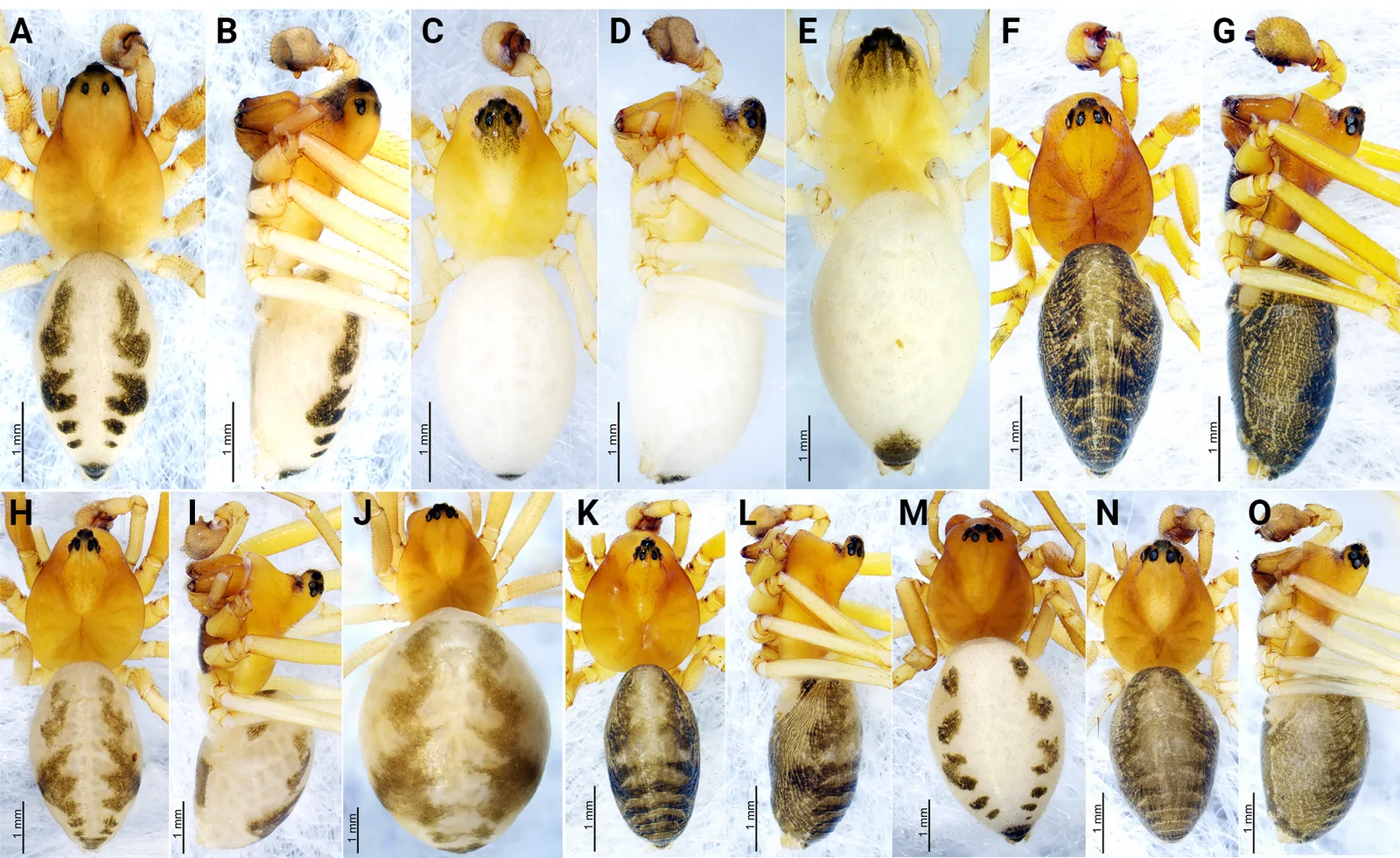
*Shaanxinus baitianhe* sp. nov. (male holotype **A, B**); *S. griseolineatus* (Schenkel, 1936) (male **C, D**; female **E**), *S. kuankuoshui* sp. nov. (male holotype **F, G**); *S. liangdang* sp. nov. (male holotype **H, I**; female paratype **J**); *S. micangshan* sp. nov. (male holotype **K, L**; female paratype **M**), and *S. zhongzhai* sp. nov. (male holotype **N, O**). **A**, **C, E, F, H, J, K, M, N**. Habitus, dorsal view; **B, D, G, I, L, O**. Habitus, lateral view.

**Figure 11.**
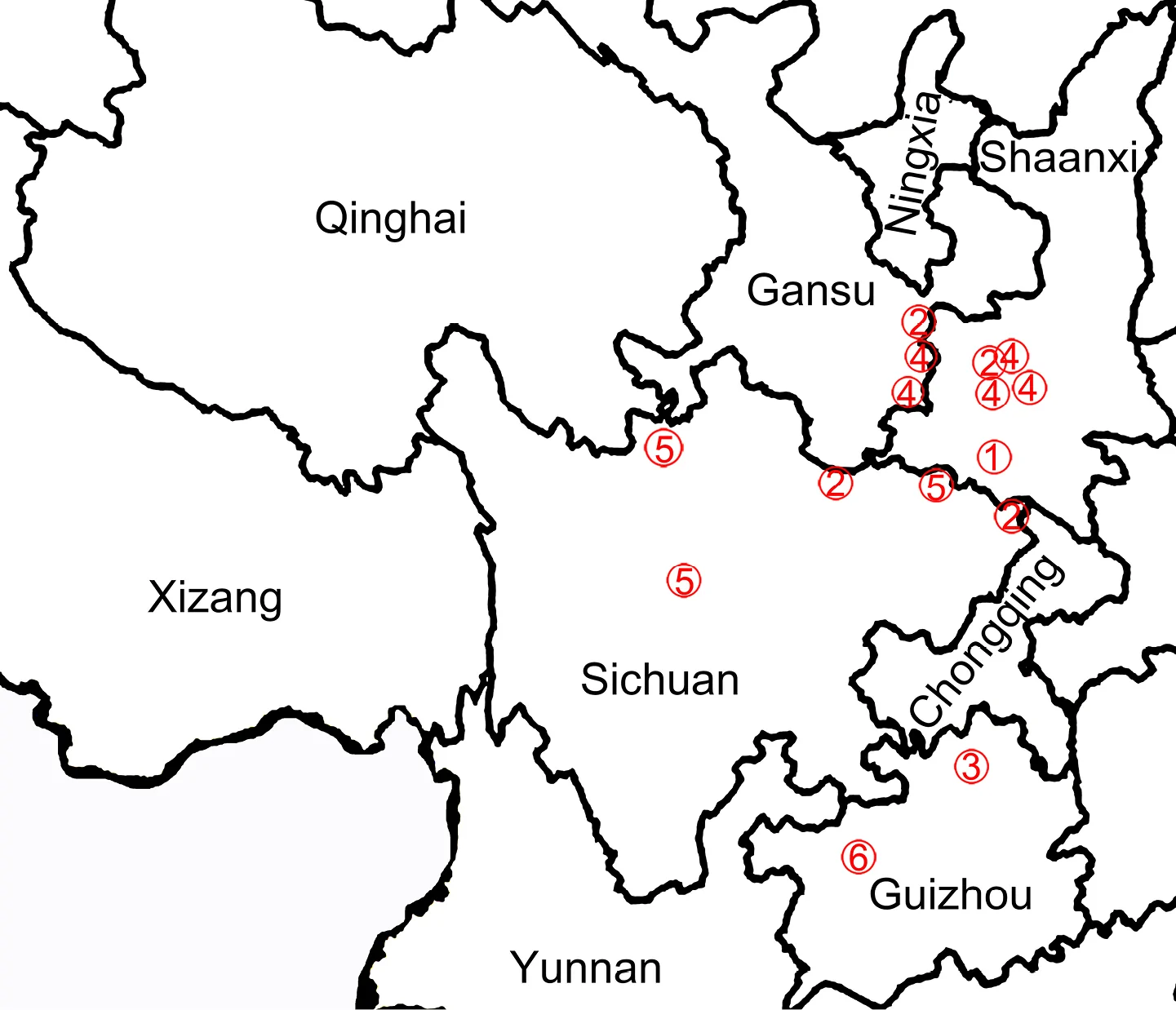
Distribution of *Shaanxinus* species documented in this study: 1. *S. baitianhe* sp. nov. (Shaanxi), 2. *S. griseolineatus* (Schenkel, 1936) (Gansu, Shaanxi and Sichuan), 3. *S. kuankuoshui* sp. nov. (Guizhou), 4. *S. liangdang* sp. nov. (Gansu and Shaanxi), 5. *S. micangshan* sp. nov. (Sichuan), and 6. *S. zhongzhai* sp. nov. (Guizhou).

Figs 9A–D, 10N, 10O, 11

##### Chinese name.

中寨陕蛛.

##### Type material.

***Holotype***: • 1♂, China: Guizhou Province, Bijie City, Nayong County, Yongxi Town, Zhongzhai Village, 26°41.196'N, 105° 22.802'E, alt. 1691 m, 2012-7-2, Wang Dong, Jiang Xuankong leg. (SWUC-T-LIN-44-01).

##### Etymology.

The specific epithet is derived from the type locality.

##### Diagnosis.

Male of *Shaanxinus zhongzhai* sp. nov. can be distinguished from all other congeners by the distal arm of the paracymbium tip with several small teeth; protegulum reduced; tip of lateral arm of radix forming a perpendicular angle (Fig. 9B).

##### Description.

**Male** (holotype, Fig. 10N, O): total length: 2.26. Carapace 1.04 long, 0.83 wide, yellow, cephalic lobe 0.48 high with many microsetae; sulci present below anterior eye row; fovea, cervical and radial grooves distinct. Clypeus 0.24 high. Chelicerae with 4 promarginal and 4 retromarginal teeth. AER recurved, PER straight, wider than AER. Eye sizes and interdistances: AME 0.05, ALE 0.07, PME 0.06, PLE 0.07, AME–AME 0.06, PME–PME 0.08, AME–PME 0.11, AME–ALE 0.05, PME–PLE 0.05, ALE–ALE 0.31, PLE–PLE 0.34, ALE–PLE 0.01. Length of legs: I 3.75 (1.05, 1.25, 0.91, 0.54), II 3.94 (1.12, 1.28, 0.97, 0.58), III 2.76 (0.84, 0.83, 0.69, 0.40), IV 3.19 (0.91, 0.97, 0.84, 0.47). Leg II longer than I. TmI 0.21. Tibial spine formula: 2-2-2-2. Abdomen 1.34 long, 0.83 wide, oval, grey, with black pattern followed by 5 transverse chevrons, ventral side grey.

Pedipalp (Fig. 9A–D). Patella slightly shorter than tibia, ventrally grooved. Tibia with 2 retrolateral and 1 dorsal trichobothria, posterior margin with long, thick spines. Paracymbium C-shaped, distal arm tip with depression, margin with several small teeth in retrolateral view. Tegulum retrolaterally extending, with small reduced membranous protegulum. Distal suprategular apophysis disc-shaped, with bifurcated end. Radix with rod-like anterior radical process, with pointed end; lateral extension of radix slightly curved, apical end with large projection in retrolateral view; tailpiece small, triangular, with pointed end; median membrane present; embolus arc-shaped, terminal part ends in apex of lateral extension of radix.

**Female**. Unknown.

##### Distribution.

Known only from the type locality (Fig. 11).

## Discussion

The addition of five new species described here brings the total number of *Shaanxinus* species to 26, substantially expanding the known distribution of the genus beyond its previous records in China (Shaanxi, Hebei, and Taiwan), as well as Russia (Far East) and Vietnam (WSC 2026). The discovery of five new species from Gansu, Guizhou, Shaanxi, and Sichuan Provinces underscores that the species diversity of *Shaanxinus* in continental East Asia remains significantly underestimated, consistent with the conclusions of Lin et al. (2019), who emphasised that more intensive collecting efforts are needed in this region.

This study builds on this revision by describing five new species from Gansu, Shaanxi, Sichuan, and Guizhou and examining additional material of *S. griseolineatus*. Geographically, these records improve coverage of mainland mountain regions and complement the Taiwanese and Vietnamese material that Lin et al. (2019) treated. Morphologically, the new species document additional variation in male pedipalp structures and female epigynes. Comparisons with previously described species provide diagnostic combinations for recognising the new taxa and broaden the morphological evidence available for future revision of the genus. These findings demonstrate that diversity in mainland *Shaanxinus* remains incompletely documented and provide a basis for subsequent sampling and phylogenetic investigation.

## Supplementary Material

XML Treatment for Shaanxinus

XML Treatment for Shaanxinus baitianhe

XML Treatment for Shaanxinus griseolineatus

XML Treatment for Shaanxinus kuankuoshui

XML Treatment for Shaanxinus liangdang

XML Treatment for Shaanxinus micangshan

XML Treatment for Shaanxinus zhongzhai

## Additional information

### Artificial Intelligence (AI) use

The authors accept full responsibility for the content of the manuscript, including the disclosure of any use of AI.

No AI tools were used in the preparation of this manuscript.

### Author ORCIDs

M. Irfan https://orcid.org/0000-0003-0445-9612

T. Rehman https://orcid.org/0009-0002-0617-3589

L.-Y. Wang https://orcid.org/0000-0002-5250-3473

Z.-S. Zhang https://orcid.org/0000-0002-9304-1789

### Data availability

All of the data that support the findings of this study are available in the main text.
